# DNA polymerases drive DNA sequencing-by-synthesis technologies: both past and present

**DOI:** 10.3389/fmicb.2014.00305

**Published:** 2014-06-24

**Authors:** Cheng-Yao Chen

**Affiliations:** Protein Engineering Group, IlluminaSan Diego, CA, USA

**Keywords:** Sanger sequencing, chain terminators, reversible terminators, sequencing-by-synthesis, DNA polymerase, next-generation sequencing, protein engineering

## Abstract

Next-generation sequencing (NGS) technologies have revolutionized modern biological and biomedical research. The engines responsible for this innovation are DNA polymerases; they catalyze the biochemical reaction for deriving template sequence information. In fact, DNA polymerase has been a cornerstone of DNA sequencing from the very beginning. *Escherichia coli* DNA polymerase I proteolytic (Klenow) fragment was originally utilized in Sanger’s dideoxy chain-terminating DNA sequencing chemistry. From these humble beginnings followed an explosion of organism-specific, genome sequence information accessible via public database. Family A/B DNA polymerases from mesophilic/thermophilic bacteria/archaea were modified and tested in today’s standard capillary electrophoresis (CE) and NGS sequencing platforms. These enzymes were selected for their efficient incorporation of bulky dye-terminator and reversible dye-terminator nucleotides respectively. Third generation, real-time single molecule sequencing platform requires slightly different enzyme properties. Enterobacterial phage ϕ29 DNA polymerase copies long stretches of DNA and possesses a unique capability to efficiently incorporate terminal phosphate-labeled nucleoside polyphosphates. Furthermore, ϕ29 enzyme has also been utilized in emerging DNA sequencing technologies including nanopore-, and protein-transistor-based sequencing. DNA polymerase is, and will continue to be, a crucial component of sequencing technologies.

## INTRODUCTION

Since the advent of enzymatic dideoxy-DNA sequencing by Frederic Sanger (Sanger et al., 1977), sequencing DNA/RNA has become standard practice in most molecular biology research. The proliferation of next-generation sequencing (NGS) technologies has further transformed modern biological and biomedical research. Today, large-scale whole genome sequencing has become routine in life science research. Although technical advances in current NGS technologies have dramatically changed the way nucleic acids are sequenced, the engine ultimately responsible for these modern innovations remains unchanged. Like Sanger sequencing, today’s NGS technologies, with the exception of oligonucleotide-based ligation sequencing (Drmanac et al., 2010), still require a DNA polymerase to carry out the necessary biochemical reaction for replicating template sequence information. This unique, polymerase-dependent sequencing approach is generally referred to as DNA sequencing-by-synthesis (SBS), because the consecutive sequencing reaction concurrently generates a newly synthesized DNA strand as a result.

However, unlike Sanger sequencing, DNA polymerases utilized in NGS technologies are more diverse and tailor-made. The Klenow enzyme, a proteolytic fragment of *Escherichia coli* DNA polymerase I. was originally utilized in Sanger’s dideoxy chain-terminating DNA sequencing chemistry (Sanger et al., 1977). This enzyme was chosen for its efficient incorporation of 2′, 3′-dideoxynucleotides (ddNTPs) that leads to chain termination of DNA synthesis (Atkinson et al., 1969). From this humble beginning, followed by a robust sequencing chemistry improvement, the nucleotide substrates used for DNA sequencing became larger and bulkier. First, four fluorescent dyes with distinct, non-overlapping optical spectra were attached to either purine or pyrimidine bases, respectively, and even the terminal gamma phosphate of four (A, T, C, and G) nucleotides for the ease of signal detection (Smith et al., 1986; Ju et al., 2006; Guo et al., 2008, 2010; Eid et al., 2009; Korlach et al., 2010). Second, the 3′ hydroxyl group on deoxyribose of four nucleotides was replaced with a larger, cleavable chemical group used to reversibly terminate DNA synthesis (Ju et al., 2006; Guo et al., 2008, 2010). As a result, the original Klenow enzyme no longer efficiently incorporated these newly modified nucleotides. DNA polymerases with different enzymatic properties were required for improving the nucleotide incorporation reactions. Fortunately, the adoption of NGS sequencing in life science research allowed a rapid expansion of organism-specific, genome sequence information accessible via public database. Various DNA polymerases from mesophilic/thermophilic viruses, bacteria, and archaea were discovered and later screened for efficient incorporation of modified nucleotides in new DNA sequencing methods. A pool of new, advantageous DNA polymerases from a wide variety of microorganisms were selected and served as protein backbones for further improvement via protein engineering or directed enzyme evolution (Patel and Loeb, 2001). Evolved DNA polymerases with improved biochemical performances were ultimately utilized for each, unique sequencing technology.

This article briefly covers (1) the progression of decades’ enzymatic DNA sequencing methods reliant on functions of DNA polymerase for synthesizing new DNA strands; (2) the novel properties of DNA polymerase that are required for high-precision DNA sequencing; (3) the influence of nucleotide modifications on DNA polymerase research that ultimately lead to improved sequencing performance; (4) the application of DNA polymerases in emerging DNA sequencing methods. Readers interest in learning more about other sequencing methods can refer to these literatures (Landegren et al., 1988; Ding et al., 2012) for more information.

## OVERVIEW OF DNA POLYMERASE FAMILIES AND FUNCTIONS

Since the discovery of DNA polymerase I in *E. coli* by Arthur Kornberg’s group in the late 1950s (Lehman et al., 1958a,b), multiple DNA polymerases have been discovered, purified and characterized from bacteria, eukaryotes, archaea, and their viruses. The expansion of organism-specific, genome sequence information accessible via public database, together with advanced search-algorithms based on DNA polymerase structure–function relationships, have reduced the time necessary for identification of additional, putative DNA polymerases from a variety of sources (Burgers et al., 2001). Based on the phylogenetic relationships of *E. coli* and human DNA polymerases, DNA polymerases are generally classified into seven main families: A (*E. coli* Pol I), B (*E. coli* Pol II), C (*E. coli* Pol III), D, X (human Pol β-like), Y (*E. coli* Pol IV and V and TLS polymerases), and RT (reverse transcriptase) (Burgers et al., 2001; Langhorst et al., 2012). All living organisms, except viruses, harbor multiple types of DNA polymerases for cellular functions. Interestingly, neither bacteria, eukaryotes nor archaea contain all families of DNA polymerases. As summarized in **Table 1**, the family C DNA polymerases are unique to bacteria, and have not been found in either eukaryotes or archaea (Hübscher et al., 2010; Langhorst et al., 2012). Likewise, the family D polymerases are restricted to archaea (Euryarchaeota), and do not exist in bacteria or eukaryotes (Hübscher et al., 2010; Langhorst et al., 2012). Another characteristic exclusive to archaeal DNA polymerases is the presence of intervening sequences (inteins) within the polymerase coding genes (Perler et al., 1992). These inteins cause in-frame insertions in archaeal DNA pols and must be spliced out in order to form mature enzymes (Hodges et al., 1992).

**Table 1:** Families and properties of cellular DNA replicases (Kunkel, 2004; Hübscher etal., 2010; Greenough etal., 2014).

Polymerase family	Bacteria (*E. coli*)	Eukaryotes (human)	Archaea	Viruses	3′ to 5′ exonuclease activity	**Error rate (fidelity)	Enzymes used in assays
A	Pol I [*pol A*]	Pol γ (p140/p55/p55) Pol θ(p100/p90/p80) Pol ν	N.A.	T3, T5, T7	Yes	~10^-^^5^–10^-^^7^	Klenow, Klen*Taq*, *Taq*, *Bst*, *Bsu*, T7
B	Pol II [*pol B*]	Pol α/primase (p180/p68/Pri2/Pri1) Pol δ (p125/p66/p50/p12) Pol ε (p260/p59/p17/p12) Pol ζ (p350/p24)	Pol BI Pol BII Pol BIII	HSV-1, RB69, T4, ϕ29	Yes	~10^-^^6^	T4, ϕ29, *9°N, KOD1, Pfu, Vent*
C	Pol III [*pol C*] core (α/ε/θ)	N.A.	N.A.	N.A.	Yes	~10^-^^6^	N.A.
D	*N.A.	N.A.	Pol D (DP2/DP1)	N.A.	Yes	10^-^^4^–10^-^^5^	N.A.

In the bacterial column, the gene for each corresponding protein is indicated in the bracket. In the eukaryotic and archaeal column, the components of each holoenzyme are listed in the parentheses. *N.A. denotes “not applicable.” **The unit for “Error Rate” is one error per incorporated base.

The basic function of DNA polymerases (cellular DNA replicases) are to faithfully replicate the organism’s whole genome and pass down the correct genetic information to future generations. In bacteria, family C DNA polymerases, such as Pol III holoenzyme in *E. coli* or *Bacillus subtilis*, are the key element for driving chromosomal replication and thus absolutely mandatory for cell viability (Gefter et al., 1971; Nusslein et al., 1971; Gass and Cozzarelli, 1973, 1974). Besides the Pol III holoenzyme, the A-family Pol I also participates in bacterial DNA replication (Olivera and Bonhoeffer, 1974). Pol I contains a separate 5′ to 3′ exonuclease, independent of the DNA polymerase domain, that can remove RNA primers and concurrently fill in the nucleotide gaps between Okazaki fragments during lagging strand DNA synthesis (Okazaki et al., 1971; Konrad and Lehman, 1974; Xu et al., 1997). Unlike bacterial cells, eukaryotic B-family DNA polymerases, such as Pol δ and ε in human and yeast, are responsible for nuclear chromosomal replication (Miyabe et al., 2011). Recent studies in yeast by Thomas Kunkel’s group suggest that Pol δ and ε divide their roles during DNA replication and are responsible for lagging and leading strand DNA synthesis, respectively (Pursell et al., 2007; Kunkel and Burgers, 2008; Nick McElhinny et al., 2008; Miyabe et al., 2011). In archaea, both B- and D-family pols are involved in genomic replication. However, the role of each Pol *in vivo* remains controversial. From biochemical studies, both Pol B and D enzymes from hyperthermophilic *Pyrococcus abyssi* are proposed to function together in DNA replication (Henneke et al., 2005). In contrast, a recent genetic study in *Thermococcus kodakarensis* showed that Pol D alone is sufficient for cell viability and genomic replication which argues that Pol D, rather than Pol B, is the main replicative DNA polymerase in this archaeon (Cubonova et al., 2013). It is possible that the requirements for Pol B and D enzymes in DNA replication are different in separate phyla of Archaea.

In summary, all DNA polymerases engaged in cellular genome replication, regardless of origin, have the following common features (See **Table 1**): (1) they appear to form a multi-subunit enzyme complex (holoenzyme); (2) they all possess an intrinsic 3′ to 5′ exonuclease, or proofreading activity, that removes misincorporated nucleotides immediately after nucleotide incorporation to ensure high-fidelity of DNA synthesis (**Figure 3A**). In contrast to the major cellular DNA polymerases, functions of X, Y, and RT families of Pols are more diverse and specialized in many DNA processes, such as DNA repair, translesion synthesis, and eukaryotic telomere maintenance (Hübscher et al., 2010). None of these Pols have any intrinsic 3′ to 5′ proofreading exonuclease activity and are thus more error-prone during DNA synthesis (Kunkel and Bebenek, 2000; Kunkel, 2004, 2009).

## CHOOSING THE RIGHT DNA POLYMERASE FOR DNA SEQUENCING

**FIGURE 1:**
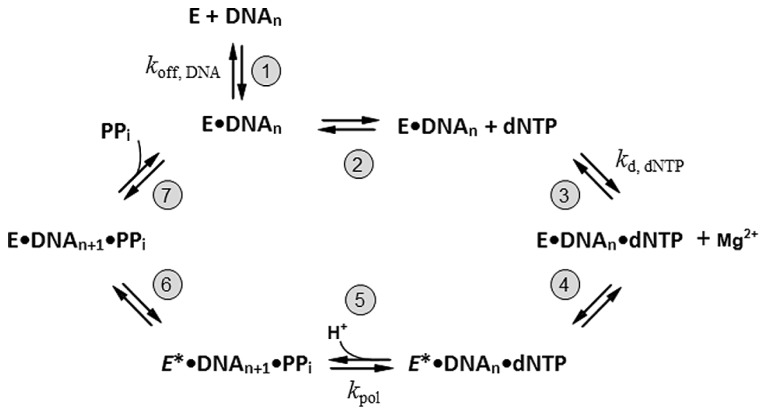
**The minimal catalytic steps required for single-nucleotide incorporation by DNA polymerase.** The addition of nucleotide to the 3′ end of a primer by DNA polymerase passes through a temporally ordered mechanism. The reaction begins with the binding of free DNA polymerase (E) to a duplex primer/template DNA complex (DNA_n_) resulting in a binary enzyme-DNA complex (E•DNA_n_; step 1). The *k*_off,_ _DNA_ represents the rate of enzyme dissociation from the E•DNA_n_ complex. Addition of the correct nucleotide (dNTP) in the presence of divalent cations, such as Mg^2^^+^, promotes the enzyme-DNA-dNTP ternary complex formation (E•DNA_n_•dNTP; step 2 and 3). The *k*_d,__dNTP_ denotes the nucleotide binding constant of the enzyme. The binding of the dNTP induces the first conformational change of the enzyme in the ternary complex (E*•DNA_n_•dNTP; step 4; Wong et al., 1991). The actual chemistry happens (step 5). The phosphodiester bond is formed between the α-phosphate of the incoming dNTP and 3′-OH of the primer terminus and produces an added nucleotide base to the primer terminus (DNA_n__+__1_). The chemical reaction generates a pyrophosphate (PP_i_) and proton molecule (H^+^). This is followed by a second conformational change of the enzyme (step 6), which allows the final release of the PP_i_ leaving group (step 7). The nucleotide incorporation cycle is complete after PPi release. If the enzyme remains associated with DNA, a new round of nucleotide addition will continue until the enzyme dissociates from the DNA (processive synthesis).

Growing numbers of DNA polymerases, each with distinct functions, provide abundant enzymatic resources for improving current and emerging DNA sequencing techniques. However, not all families of DNA polymerases are suitable for high-precision DNA sequencing reactions. To be considered, and ultimately applied for a particular method of sequencing, the DNA polymerase should possess the following properties:

(1) The pol has to be a DNA-dependent DNA polymerase. Some X and RT-family enzymes do not require a DNA template for replication and are thus not suitable for DNA sequencing; for instance, X-family terminal deoxynucleotidyl transferases (Tdt) are template-independent DNA polymerases which catalyze the addition of deoxynucleotides (dNTPs) to the 3′-OH ends of DNA in the absence of a DNA template (Kato et al., 1967; Coleman et al., 1974). Similarly, RT-family eukaryotic telomerases are ribonucleoproteins which utilize their own, endogenous RNA template for elongation at the telomeric DNA ends (Morin, 1989; Blackburn et al., 2006). These enzymes bypass the requirement of a DNA template to function and cannot be used for DNA sequencing.
(2) The pol should rapidly incorporate nucleotides. Despite the diverse functions among DNA polymerases, the basic mechanism of nucleotide incorporation remains relatively fixed. All replicative DNA pols require a duplex primer-template DNA with a free 3′-OH group at the primer terminus, all four nucleoside triphosphates (dATP, dTTP, dCTP, and dGTP), and catalytic, divalent cations (Mg^2^^+^ or Mn^2^^+^, etc.) for the sequencing reaction. Addition of nucleotides to the 3’ end of a primer by DNA pols proceeds through a highly ordered, temporal mechanism. The minimal catalytic mechanism of single-nucleotide incorporation by DNA pol has been proposed (Donlin et al., 1991; Johnson, 1993) and is illustrated in **Figure 1**. A brief description for each reaction step can be found in the figure legend. As shown in **Figure 1**, the nucleotide incorporation efficiency (specificity) of a DNA polymerase (*k*_pol_/k_d,dNTP_) is determined by the rate of phosphodiester bond formation (*k*_pol_) and the binding constant for the cognate nucleotide (*k*_d,dNTP_; Wong et al., 1991; Johnson, 1993). DNA pols with a faster nucleotide incorporation rate and lower *k*_d,dNTP_ (large *k*_pol_ and small *k*_d,dNTP_) can catalyze DNA synthesis much more efficiently. In this aspect, none of the X and Y-family pols can meet this requirement. Both X and Y-family Pols have much lower nucleotide incorporation efficiency (Brown et al., 2011a,b) compared to cellular DNA replicases from A, B, C, or D-family enzymes (Patel et al., 1991; Wong et al., 1991; Bloom et al., 1997; Zhang et al., 2009). Therefore, they are not ideal for DNA sequencing.
(3) The pol must have high replicative fidelity to minimize systematic sequencing errors. In order to accurately read DNA template sequence information, the DNA pol must faithfully incorporate the correct, matched nucleotides along the DNA template. The fidelity of nucleotide incorporation by X, Y, and RT Pols range from ~10^-^^1^ to 10^-^^4^ error per base incorporated, two to three orders of magnitude lower than high-fidelity cellular DNA polymerases from A, B, or C-family enzymes (Kunkel, 2004). These repair pols generally make errors during DNA synthesis (Kunkel and Bebenek, 2000; Kunkel, 2004, 2009) and are not appropriate for high-precision DNA sequencing applications.
(4) The pol should possess long, intrinsic, replicative processivity. The processivity of DNA polymerase is defined as the number of dNTPs incorporated during complex formation with a primer/template (P/T) DNA. As illustrated in **Figure 1**, the processivity of DNA pol is directly related to two parameters: (1) the rate of dNTP incorporation by the enzyme (*k*_pol_ of step 5); (2) the enzyme’s dissociation rate from the enzyme–DNA binary complex (*k*_off,DNA_ of step 1). Under these parameters, the enzyme remains associated with the template DNA, it carries out sequential rounds of nucleotide incorporation until it dissociates from the binary complex (**Figure 1**, steps 2–7). Theoretically, processivity of the DNA polymerase can be estimated by calculating the ratio of *k*_pol_/*k*_off_, _DNA_. Amongst DNA polymerases, only viral DNA polymerases have unusually intrinsic, long processivity. For instance, the enterobacterial phage ϕ29 DNA polymerase (a B-family enzyme) possesses intrinsic, long, replicative processivity and can replicate its own genomic DNA (~18,000 base pairs) *in vitro* without any accessory cofactors (Blanco and Salas, 1985). In contrast, most cellular DNA replicases from A, B, C, and D families are distributive, and limited to only a few nucleotide incorporations. These DNA replicases must physically interact with their processivity factors, including β-sliding clamp in bacteria, and PCNA in eukaryotes and archaea, in order to achieve a long processivity during DNA replication (Johnson and O’Donnell, 2005). No X, Y, or RT-family enzymes are processive.
(5) The pol should function as a monomer for ease of protein production and further modification. As illustrated in **Table 1**, most A, B, C, and D-family DNA replicases form a multi-subunit enzyme complex (holoenzyme). Components of these replicative holoezymes are difficult to purify, and whole enzyme complexes are very challenging to reconstitute. Therefore, these types of enzyme complexes are seldom used in any DNA sequencing chemistry.

In summary, to fulfill the above requirements for high-precision DNA sequencing, only A-family enzymes from bacteria and phage viruses (such as T5 and T7 phages), and B-family pols from bacterial viruses (such as T4, Rb69, and ϕ29 phages), bacteria, and archaea (*Vent, 9°N, Pfu*, and *KOD1*) have been evaluated for sequencing chemistry development (See **Table 1**). All family A and B enzymes have an associated, intrinsic 3′ to 5′ exonuclease proofreading activity. When these enzymes incorporate an incorrect nucleotide at the primer terminus, the enzymes’ ability to extend the primer terminus diminishes, and allows the nascent DNA strand to migrate to the 3′ exonuclease site for excision (See **Figure 3A**; Donlin et al., 1991; Joyce and Steitz, 1994; Patel and Loeb, 2001). This unique partitioning mechanism of the 3′ exonuclease proofreading domain among A and B-family polymerases is disfavored for DNA sequencing. It causes asynchronous DNA sequencing reactions and generates systematic sequencing errors (**Figures 3B,C**). Therefore, the majority of A and B-family pols used for DNA sequencing are either lacking, or have an attenuated, 3′ exonuclease proofreading activity.

## NUCLEOTIDE SUBSTRATES FOR THE GENERATIONS OF DNA POLYMERASE-BASED SEQUENCING

**FIGURE 2:**
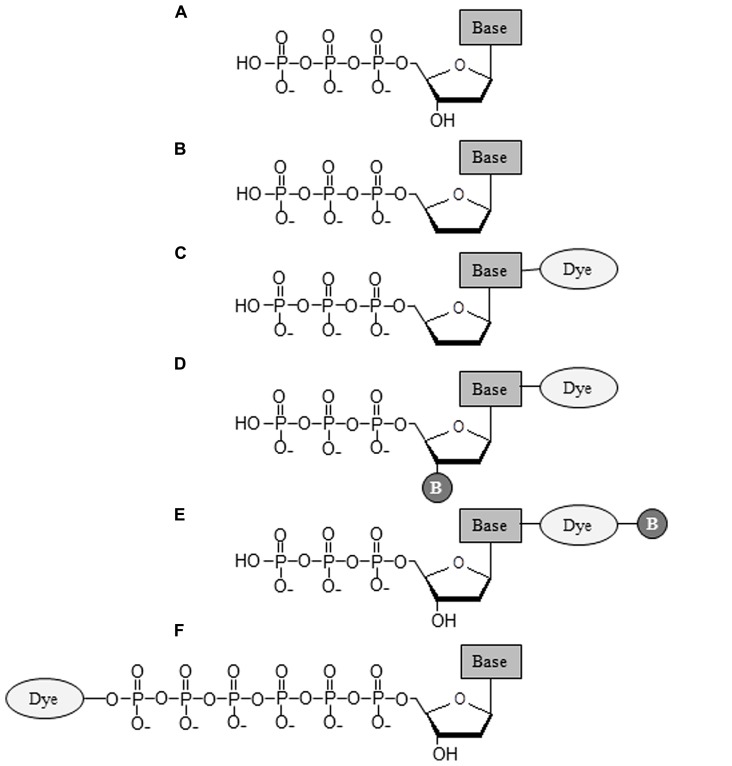
**Structures of nucleotides utilized in the generations of DNA polymerase-based sequencing methods.(A)** Deoxynucleotides (dNTPs); **(B)** 2′, 3′-dideoxynucleotides (ddNTPs); **(C)** Dye-terminators; **(D)** Reversible dye-terminators; **(E)** 3′-OH unblocked reversible dye-terminators; **(F)** Dye-labeled hexaphosphate nucleotides. The “Base” in the diagram represents an A, T, C or G base, and “B” indicates a cleavable chemical blockage group.

Generations of DNA polymerase-based sequencing methods and their corresponding commercial platforms are summarized in **Table 2**. As shown in **Table 2**, all methods require a DNA polymerase to catalyze the necessary biochemical reaction for extracting DNA sequence information. The fundamental difference amongst these technologies is the type of nucleotide substrate incorporated. The structures of these nucleotides are illustrated in **Figure 2**. More in-depth information regarding these nucleotides can be found in the following articles (Metzker et al., 1996; Lee et al., 1997; Kumar et al., 2005; Metzker, 2010; Chen et al., 2013a). From classical Sanger sequencing to modern NGS technologies, the nucleotide substrates used for sequencing have changed over time. In the original Sanger sequencing method, four 2′, 3′-ddNTPs (**Figure 2B**) are utilized (Sanger et al., 1977). Unlike normal dNTPs (**Figure 2A**), the ddNTPs lack the 3′-hydroxyl group (3′-OH), which is required for the phosphodiester bond formation between the incorporating nucleotide and primer terminus. Once ddNTPs are incorporated by the DNA polymerase, they terminate further addition of nucleotides from the primer terminus, and cease elongation of the DNA chain (Atkinson et al., 1969). Besides the utilization of ddNTPs, Sanger’s protocol requires a set of radioisotope-labeled primers in four, separate (A, T, C, and G) reactions. The resulting dideoxy-terminated DNA fragments must be analyzed side-by-side using slab gel electrophoresis while sequence information is deduced via autoradiography (Sanger et al., 1977). The procedure itself is extremely time consuming and further compounded by low data output. This makes such an approach insufficient at meeting the growing demand for high-throughput DNA sequencing.

**Table 2:** Generations of DNA polymerase-based DNA sequencing technologies.

Institution or company	Instrumentation	Sequencing methods	Nucleotide substrates	Detection from DNA polymerase reaction
MRC	Sanger sequencing	DNA chain termination and fragment analysis by gel	2′, 3′-dideoxynucleotides	Nucleotide incorporation
Applied biosystems/life technologies	ABI genetic analyzer series	DNA chain termination and fragment analysis by CE	Dye-terminators	Nucleotide incorporation
Illumina Qiagen/IBS	GA/MiSeq/HiSeq Max-Seq/Mini-20	Stepwise SBS	Reversible dye-terminators	Nucleotide incorporation
*Helicos biosciences	HeliScope	Stepwise single-molecule SBS	3′-OH unblocked reversible dye-terminators	Nucleotide incorporation
Pacific biosciences	PACBIO RS II	Real-time single-molecule SBS	γ-phosphate-labeled nucleotides	Nucleotide incorporation
Roche/454 life sciences	GS FLX/GS Junior	Sequential SBS	Nature dNTPs	PP_i_ release
Ion torrent/life technologies	Ion PGM/proton	Sequential SBS	Nature dNTPs	H^+^ release

*On November 15, 2012, Helicos Biosciences filed for Chap. 11 bankruptcy.

**FIGURE 3:**
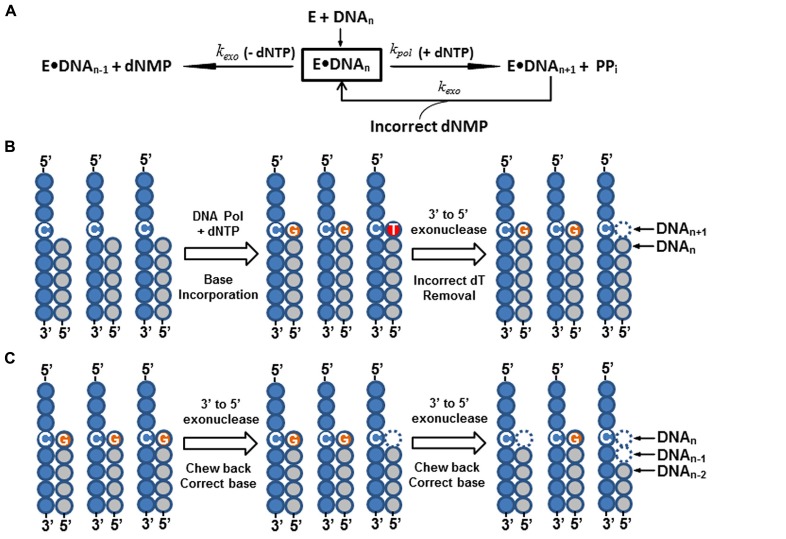
**Intrinsic 3′ to 5′ exonuclease activity of DNA polymerase and its impact on DNA sequencing reactions. (A)** A simplified kinetic model illustrating the proofreading function and nucleotide excision activity of 3′ to 5′ exonuclease of DNA polymerase (Donlin et al., 1991; Johnson, 1993). As shown in the figure, when a free DNA polymerase (E) is mixed with a duplex primer/template DNA complex (DNA_n_), they form a stable, binary enzyme-DNA complex (E•DNA_n_). In the presence of nucleotide (+dNTP) and divalent cations (Mg^2^^+^, Mn^2^^+^, etc.), the enzyme rapidly incorporates (*k*_pol_) a single-nucleotide base to the primer terminus (DNA_n__+__1_) and concurrently drives release of free pyrophosphate (PP_i_). However, when an incorrect nucleotide is misincorporated by the enzyme, it causes a base-pair mismatch at the primer terminus (DNA_n__+__1_; Panel **B**, middle cartoon, a dC:dT mismatch). This mismatched nucleotide base at the primer terminus greatly impedes the DNA polymerase’s capability to incorporate the next nucleotide base (greatly reduced *k*_pol_ value) and triggers a rapid transfer of DNA primer strand to the intrinsic 3′ to 5′ exonuclease domain. The mismatched nucleotide base is then removed (incorrect deoxynucleoside monophosphate, dNMP) by the 3′ to 5′ exonuclease (*k*_exo_). Once the mismatched nucleotide base is excised by the 3′ to 5′ exonuclease, the corrected primer strand is shifted back to the DNA polymerase catalytic domain (E•DNA_n_). As a result, the misincorporated nucleotide is removed and the enzyme is ready to incorporate the correct nucleotide (see Panel **B**, left to right cartoons). In addition to the base-mismatched proofreading function of the 3′ to 5′ exonuclease domain, it will also gradually chew back the primer strand (DNA_n-1_, DNA_n-2,_ etc.) and release dNMPs in the absence of nucleotide (-dNTP; see Panel **C**, left to right cartoons). An asynchronous DNA sequencing reaction occurs when the sequencing DNA polymerase misincorporates a nucleotide base (Panel **B**), or the DNA sequencing primer is chewed back by the enzyme’s 3′ to 5′ exonuclease (Panel **C**). The outcome of both reactions produces a non-uniform duplex primer-template DNA for DNA sequencing (Panels **B,C**, the right cartoons), and causes systematic DNA sequencing errors. In the panels **B,C**, each filled circle indicates a nucleotide base. A string of filled-gray circles represents the primer strand, and a string of filled-blue circles is the template DNA strand. Specific bases (dC, dG, and dT) are indicated inside the circles.

To simplify and subsequently automate Sanger’s method, Leroy Hood’s group, then at California Institute of Technology, invented the first fluorescent sequencing (dye-primer) method based on Sanger’s approach (Smith et al., 1986). In Hood’s revised protocol, the primers used for sequencing reactions are covalently attached to four distinct colors of fluorophores at the 5′-end, corresponding to each of the A, T, C, and G reactions in Sanger sequencing. The advantages to this approach are (1) the four reaction mixtures can be combined and analyzed in a single sequencing lane; (2) the results can be directly monitored by a computer-aided fluorescence detection system, specifically matched to the emission spectra of the four dyes. These advantages allow DNA sequence information to be analyzed automatically by the computer.

Hood’s dye-primer method simplifies traditional Sanger sequencing processes but it is not, however, completely ideal for fully automated DNA sequencing, mainly due to the four, separate reactions still required. To solve this problem, the fluorescently labeled chain-terminating ddNTPs (dye-terminators) were soon introduced by Prober et al. (1987) from DuPont. Similar to the dye-primers, a set of fluorescently distinguished fluorophores are covalently attached to each of four ddNTPs (See **Figure 2C**). Adaptation of dye-terminators for Sanger sequencing workflow makes the four, base-specific chain termination reactions happen in one, single reaction tube. DNA polymerase is able to simultaneously incorporate four dye-terminators and generate the terminated DNA pieces for sequence analysis (Rosenthal and Charnock-Jones, 1992, 1993). The speed and throughput of dye-terminator sequencing was drastically improved when the automated capillary-array electrophoresis (CAE) was adopted for DNA analysis (Drossman et al., 1990; Luckey et al., 1990; Zagursky and McCormick, 1990; Dovichi and Zhang, 2000).

The dye-terminator-CE method has greatly improved sequencing performance and has become the laboratory standard for DNA sequencing over the past few decades. However, the technique itself is still very limited, especially for large-scale, whole genome sequencing. Increasing the sequencing throughput of dye-terminator-CE chemistry requires additional capillary tubes to be implemented. This becomes impractical for the application of high-throughput, multiplexing sequencing that is capable of sequencing millions of different DNA strands concurrently. To alleviate this limitation, reversible dye-terminators were introduced to the modified, dye-terminating sequencing scheme. Similar to dye-terminators (**Figure 2C**), reversible dye-terminators (**Figure 2D**) are also missing the 3′-OH group needed for DNA polymerase extension of the primer terminus. Incorporation of these modified nucleotides by DNA polymerase terminates DNA chain elongation (Bentley et al., 2008; Guo et al., 2008; Hutter et al., 2010). When these reversible dye-terminators are used in parallel with immobilization of DNA molecules on a solid-state surface, the individual DNA sequence can be directly ascertained from the base-specific, terminated DNA molecules recognized by the fluorescent imaging system (Bentley et al., 2008; Guo et al., 2008, 2010). As a result, the requirements for capillary electrophoresis (CE) analysis in a typical dye-terminator approach are no longer necessary, and millions of different DNA molecules can be sequenced simultaneously. Differentiating themselves from dye-terminators, reversible dye-terminators contain cleavable chemical groups at the 3′ position of the pentose and linker region, located between the base and attached fluorophore (**Figure 2D**; Bentley et al., 2008; Guo et al., 2008; Hutter et al., 2010). These cleavable chemical groups can be removed in order to restore the normal 3′-OH group of deoxyribose and maintain the integrity of bases attached with dye. DNA chains can thus be further extended by the DNA polymerase and incorporation can resume once more in the next reaction cycle (Bentley et al., 2008; Guo et al., 2008, 2010). A similar sequencing scheme was also carried out using another class of reversible dye-terminators with normal 3′-OH groups (Wu et al., 2007; Pushkarev et al., 2009; Litosh et al., 2011; Gardner et al., 2012). These 3′ unblocked, reversible terminators possess both chemical blockage group and fluorescent dye attached to the same base (**Figure 2E**), and can be removed by either chemical cleavage or UV light (Pushkarev et al., 2009; Litosh et al., 2011).

In both classes of reversible dye-terminators, cleavage of the linker group carrying the fluorescent dye leaves extra chemical molecules on the normal purine and pyrimidine bases. These molecular remnants may perturb the protein–DNA interaction and eventually impact the sequencing performance of the DNA polymerase (Metzker, 2010; Chen et al., 2013a). To circumvent this concern, terminal γ-phosphate, fluorescently labeled nucleoside polyphosphates (**Figure 2F**) were developed for the more advanced, third-generation DNA sequencing technique (Kumar et al., 2005; Korlach et al., 2010). There are two major advantages of performing DNA sequencing with γ-phosphate-labeled nucleotides over conventional chain terminators. First, the nucleotides, once incorporated, don’t generate a molecular scar on the newly synthesized DNA, and second, they enable real-time, single-molecule SBS (Korlach et al., 2010). Because the phosphoryl transfer reaction only occurs between the 3′-OH group of the primer terminus and α-phosphate of the incoming nucleotide, the conclusion of each enzymatic reaction results in one nucleotide addition to the primer terminus plus a pyrophosphate (PPi) leaving group (**Figure 1**, steps 5–7; Steitz, 1997, 1999). Hence, any fluorophore covalently attached to the PPi leaving group will be released after nucleotide addition to the primer terminus, and thus leave no molecular vestige in the DNA. Since the added nucleotide possesses no blockage group to hinder DNA elongation from the primer terminus, the sequencing reaction can continue uninterrupted.

Finally, there are no DNA scar issues for both pyrosequencing technology (Ronaghi et al., 1996, 1998), which detects the release of PP_i_ after nucleotide addition by DNA polymerase, and semiconductor-based proton sequencing technique (Rothberg et al., 2011), which monitors the proton (H^+^) release during phosphodiester bond formation between the 3′-OH and α-phosphate of incoming nucleotide. Both technologies utilize natural nucleoside triphosphates (dNTPs) for their sequencing reactions (**Table 2** and **Figure 2A**).

## CHALLENGES OF RAPIDLY EVOLVING NUCLEOTIDE SUBSTRATES ON DNA POLYMERASE RESEARCH

A series of nucleotide modifications, created for rapidly changing DNA polymerase-based sequencing technologies has created a daunting task for DNA polymerase researchers to look for, design or evolve compatible enzymes for ever-changing DNA sequencing chemistries. From the beginning, A-family *E. coli* DNA polymerase I (Pol I) or its proteolytic (Klenow) fragment was chosen by Dr. Sanger for his dideoxy-sequencing chemistry (Sanger et al., 1977). This was the only DNA polymerase available at the time and, quite fortunately, tolerated incorporation of 2′, 3′-ddNTPs (Atkinson et al., 1969). However, Pol I effectively discriminates between a deoxy- and dideoxyribose in the nucleoside triphosphate, and does not incorporate ddNTPs very well (Atkinson et al., 1969). In fact, the incorporation rate of ddNTP by Pol I is several hundred-fold slower than that of normal dNTPs and is also sequence context-dependent (Tabor and Richardson, 1989). This sequence-specific ddNTP incorporation by Pol I creates non-uniform band intensities on the sequencing gel. This phenomenon becomes increasingly problematic, especially in the dye-primer/terminator sequencing, because the method of sequence information retrieval relies on the interpretation of fluorescent intensity of each dideoxy-terminated DNA band from the gel or capillary tubes. Similar results were reported with thermostable, Family A, *Thermus aquaticus* (*Taq*) DNA polymerase I (Innis et al., 1988).

In contrast, phage T7 DNA polymerase does not distinguish ddNTPs from dNTPs, and incorporates both types of nucleotides at nearly equal efficiencies (Tabor and Richardson, 1987; Brandis et al., 1996). Thus, the intensities of dideoxy-terminated bands are significantly more uniform with T7 pol in Sanger sequencing. To understand the molecular basis for this discrepancy, sequence analysis and biochemical studies were conducted among these three, A-family enzymes. The results indicate that a single phenylalanine to tyrosine residue change (Y526) on T7 pol, homologous position (F672), of a highly conserved finger motif (motif B) in A-family pols greatly reduces the enzyme’s ability to select against ddNTPs (Tabor and Richardson, 1995). Biochemical studies further confirm that mutant Pol I, or *Taq,* carrying a F672Y or F667Y mutation, respectively, loses its discriminatory ability for ddNTPs, and thus incorporates ddNTPs very efficiently (Patel and Loeb, 2001). Additionally, these two mutant proteins were demonstrated to incorporate fluorescein- and rhodamine-labeled dye-terminators, three orders of magnitude more efficiently than their wild-type parent enzymes (Tabor and Richardson, 1995). Subsequently, T7, F672Y Pol I, and F667Y *Taq* pols were all used for manual and automated Sanger sequencing (Tabor and Richardson, 1987, 1989; Rosenthal and Charnock-Jones, 1992; Tabor and Richardson, 1995). However, *Taq* pol has become preferred for dye-terminator sequencing, because the enzyme has several advantages over Pol I or T7. The enzyme is more readily purified and modifiable for further improvement. It also has no intrinsic, 3′ to 5′ exonuclease proofreading activity, and is active over a broad range of temperatures (Innis et al., 1988). The thermostablility of *Taq* pol became essential for sequencing after the PCR-based “cycle sequencing” approach was introduced (Rosenthal and Charnock-Jones, 1993).

The Phe to Tyr mutation at position 667 on conserved motif B of *Taq* pol only addresses the deoxy- and dideoxyribose selectivity problem in dye-terminator sequencing. The enzyme, like Pol I, possesses bias. Uneven ddNTP incorporation results in variable DNA band intensities, and unequal peak heights in CE analysis, creating unwanted sequencing errors (Parker et al., 1995; Li et al., 1999). Kinetic analysis reveals that *Taq* pol favors ddGTP incorporation over other ddNTPs, with a much more robust nucleotide incorporation rate (*k*_pol_; Brandis et al., 1996). To investigate the cause of ddGTP bias, structural analysis of all four, ddNTP-trapped ternary complexes of the large fragment of *Taq* pol (Klentaq1) was implemented. The data reveals a selective interaction between the guanidinium side chain of arginine residue 660 (R660) and the O6/N7 atoms of the guanine base of the incoming ddGTP. Substitution of the Arg660 residue with a negatively charged aspartic acid completely eliminates preference for ddGTP incorporation. The R660D/F667Y double mutant of *Taq* pol greatly improves dye-terminator sequencing quality and accuracy (Li et al., 1999).

Although the F667Y mutation on *Taq* pol greatly improves the enzyme’s incorporation efficiency for dideoxy-dye-terminators, the improvement becomes marginal for the reversible dye-terminators, which carry larger chemical blocking groups than the normal 3′-OH at the 3′ position of deoxyribose (Bentley et al., 2008; Guo et al., 2008; Chen et al., 2010, 2013a; Hutter et al., 2010). The 3′ reversible terminating group is normally linked to the deoxyribose of the nucleotide through the oxygen atom of 3′-OH. A series of 3′-*O*-blocking groups have been developed including 3′-*O*-allyl (Ruparel et al., 2005; Wu et al., 2007), 3′-*O*-(2-nitrobenzyl) (Wu et al., 2007), and 3′-*O*-azidomethylene (Bentley et al., 2008). Serendipitously, reversible dye-terminators bearing either blockage group were found to be incorporated well by a variant of archaeal *9°N* DNA polymerase (a B-family Pol) of hyperthermophilic *Thermococcus* sp. *9°N*-*7* (Southworth et al., 1996; Ruparel et al., 2005; Ju et al., 2006; Bentley et al., 2008). The enzyme variant bearing A485L and Y409V double mutations on conserved motifs A and B, respectively, of the DNA polymerase shows enhanced preference for incorporating both acyclic and dideoxy dye-terminators over the parent enzyme (Gardner and Jack, 2002). The same mutational effects were also found in enzyme mutants possessing homologous mutations in other archaeal, B-family DNA polymerase species (Gardner and Jack, 1999; Gardner et al., 2004). Similarly, the analogous combination of mutations (P410L/A485T) at the same conserved protein regions of closely related, B-family DNA polymerase *Thermococcus* sp. JDF-3 also shows an additive effect on improving dye-terminator incorporation (Arezi et al., 2002). Furthermore, an A485L variant of *9°N* DNA pol, termed Therminator DNA polymerase commercially, was recently demonstrated to efficiently incorporate 3′-OH unblocked dye-terminators with a terminating 2-nitrobenzyl moiety attached to hydroxymethylated nucleobases (Gardner et al., 2012). Thus, mutations at these two conserved protein motifs of archaeal, B-family DNA polymerase might affect the enzyme’s selectivity and tolerance for modifications and substitutions on the deoxyribose and nucleobase.

Recently, a more rational approach was taken to search for variants of *Taq* pol that can accept new types of reversible terminators possessing a 3′-ONH_2_ blocking group (dNTP-ONH_2_; Chen et al., 2010). Using the structure-guided reconstruction of ancestral DNA sequence analysis on *Taq* pol, a library of 93 protein variants carrying different combinations of mutations were designed and screened for the ability to incorporate dNTP-ONH_2_ in primer-extension assays. One beneficial mutation (L616A) on *Taq* pol was identified. The L616A *Taq* enzyme variants incorporated both dNTP-ONH_2_ and ddNTPs faithfully and efficiently.

The path toward acquisition of a compatible DNA polymerase for incorporation of fluorescent, terminal polyphosphate-labeled nucleotides has not been so straightforward. Historically, the specificities of DNA polymerases toward γ-phosphate modified dNTPs are found to be very different, due to the various degrees of steric effects of substituted chemical groups on each enzyme’s dNTP binding pocket (Arzumanov et al., 1996; Martynov et al., 1997). For instance, a bulky 2, 4-dinitrophenyl group substitution at the γ-phosphate of dNTP is a good substrate for the RT-family AMV RT, but is not acceptable for A or B-family DNA polymerases (Alexandrova et al., 1998). Similar findings were reported with the bis-(2′-deoxynucleoside) 5′, 5′-triphosphates (Victorova et al., 1999). HIV-RT utilizes this type of γ-phosphate modified nucleotide very effectively, while *E. coli* Pol I and *Taq* pol do not. Interestingly, in the same study, both Pol I and *Taq* pol were found to incorporate the bis-(2′-deoxynucleoside) 5′, 5′-tetraphosphates more efficiently than the triphosphate analog (Victorova et al., 1999). Thus, the addition of an extra-phosphate moiety to the terminal γ-phosphate of dNTP seems to attenuate the steric effects on the enzyme. Alternatively stated, the extra phosphate spacer, linked to the terminal γ-phosphate of dNTP, makes the modified nucleotide better tolerated by the enzyme. Indeed, when nucleotide incorporation rates were evaluated with fluorescent, terminal phosphate-labeled nucleoside polyphosphates containing 3, or more, phosphates at the 5′-position of the nucleoside, the nucleotides possessing greater than three phosphates were more effective substrates for A and B-family DNA polymerases (Kumar et al., 2005). Later studies proved both dye-labeled nucleoside penta/hexaphosphates (dN5Ps and dN6P) alone can be used by enterobacterial phage ϕ29 DNA polymerase for incorporating thousands of bases in length, approaching natural dNTP rates (Korlach et al., 2008, 2010). This unique, long, replicative processivity of ϕ29 DNA pol, together with intrinsic, superior capability of incorporating dye-labeled, terminal polyphosphate nucleotides plays a key role in real-time, single-molecule SBS (Korlach et al., 2010).

## APPLICATIONS OF DNA POLYMERASE FOR EMERGING SEQUENCING TECHNOLOGIES

In contrast to current, SBS approaches, emergent DNA sequencing methods rely on unconventional applications of DNA polymerase. These techniques utilize DNA polymerase as a traditional incorporating enzyme, and alternatively as a molecular motor, responsible for controlled DNA translocation across the protein nanopore. Traditional, nanopore-based, SBS uses commercial Therminator γ DNA polymerase, a variant*9°N* DNA pol, to incorporate terminal, γ-phosphate-labeled nucleoside tetraphosphates. These modified nucleotides are coupled with four, different-length PEG-coumarin tags corresponding to base A, T, C, and G (Kumar et al., 2012). DNA sequence information can be ascertained by measuring current (*amp*) fluctuations of the orderly, released PEG-coumarin tags through the α-hemolysin nanopore following DNA polymerase incorporation. A related, but fundamentally different approach involves mutant *Mycobacterium smegmatis* porin A (MspA) nanopore, ϕ29 DNA polymerase, and natural dNTPs (Manrao et al., 2012). In this approach, the enzyme functions as both DNA replicative enzyme, and molecular motor, which control the speed of DNA translocation through the MspA nanopore.

Besides the nanopore-based sequencing approach, a protein, transistor-based sequencing method, leveraging electrical conductance measurement of ϕ29 DNA polymerase reactions has been reported (Chen et al., 2013b). Unfortunately, this study is currently called into question, and the merits of this particular method must be reevaluated (Chen et al., 2013b).

## CONCLUSION

Since the introduction of the first enzymatic DNA sequencing by Frederic Sanger in the mid-1970s, decades of scientific research on various DNA polymerases, starting with Arthur Kornberg’s enzyme discovery in the mid-1950s, have provided the basic understanding of how these enzymes function and replicate DNAs, further cementing the foundation for improving enzyme properties and applications in current, and future, DNA polymerase-based sequencing technologies. The large-scale of organism-specific, genome research reveals the intrinsic diversity and unique characteristics of DNA polymerases present in all kingdoms of life, including their viruses. Diverse DNA polymerases with distinct functions and properties provide a large pool of natural protein variants that can be tested, and later utilized, for continuously evolving sequencing-chemistries. Tailor-made protein variants designed via protein engineering or directed-enzyme evolution have created powerful protein-engines that have propelled the progression of DNA sequencing technologies over the past few decades. Without a doubt, DNA polymerase has been, and will continue to remain, a crucial component of future sequencing technologies.

## Conflict of Interest Statement

The author declares that the research was conducted in the absence of any commercial or financial relationships that could be construed as a potential conflict of interest.
